# The prognostic role of 18F-FDG PET/CT baseline quantitative metabolic parameters in peripheral T-cell lymphoma

**DOI:** 10.7150/jca.30415

**Published:** 2019-10-06

**Authors:** Jun Xia, Hua-Yuan Zhu, Jin-Hua Liang, Chong-Yang Ding, Li Wang, Wei Wu, Lei Cao, Tian-Lv Li, Jian-Yong Li, Wei Xu

**Affiliations:** 1 Department of Hematology, the First Affiliated Hospital of Nanjing Medical University, Jiangsu Province Hospital, Nanjing 210029, China; Key Laboratory of Hematology of Nanjing Medical University, Nanjing 210029, China; Collaborative Innovation Center for Cancer Personalized Medicine, Nanjing 210029, China; 2 Department of Nuclear Medicine, the First Affiliated Hospital of Nanjing Medical University, Jiangsu Province Hospital, Nanjing, China; 3 Department of Hematology, Affiliated Wuxi People's Hospital, Nanjing Medical University, Wuxi, China

**Keywords:** peripheral T-cell lymphoma, prognosis, the maximum of standard uptake value, total metabolic tumor volume, total lesion glycolysis

## Abstract

**Objectives:** The aim of this study is to investigate the prognostic significance of baseline maximum standard uptake value (SUVmax), whole body SUVmax (WBSUVmax), whole body metabolic tumor volume (WBMTV) and whole body total lesion glycolysis (WBTLG) in patients with peripheral T-cell lymphoma (PTCL).

**Methods:** Eighty patients with PTCL who underwent pretreatment ^18^F-PET/CT were enrolled in this study. WBMTV and WBTLG were computed by using the margin threshold of SUV>3.0. WBSUVmax was obtained by summing of SUVmax of the whole-body SUVmax of 11 nodal and 10 extra-nodal lesions.

**Results**: Median SUVmax was 13.8 (range, 4.6-35.5), median WBSUVmax was 24.6 (range, 4.6-153.4), median WBMTV was 149 cm^3^ (range, 4-4545 cm^3^) and median WBTLG was 1017 (range, 16.5-23739). Six patients with anaplastic large cell lymphoma, ALK positive were excluded in the following statistical analysis for their unique pathological types and good prognosis. The receiver operating curve (ROC) analysis showed that the optimal cut-off values of WBSUVmax, WBMTV and WBTLG with overall survival (OS) were 22.2, 169.5 cm^3^ and 746.1, respectively. Patients with high WBSUVmax, WBMTV and WBTLG had a poor prognosis. WBSUVmax, WBMTV and WBTLG were associated with international prognostic index (IPI) and prognostic index for T-cell lymphoma (PIT). In multivariate analysis, WBTLG and PIT were independent prognostic factors of both progression free survival (PFS) and OS.

**Conclusions**: Our study shows that high WBTLG, WBMTV and WBSUVmax could predict a relatively poor prognosis, and has a highly significant association with PIT and IPI.WBTLG could be an independent predictive factor for survival outcomes in patients with PTCL.

## Introduction

Peripheral T-cell lymphoma (PTCL) is a clinically heterogeneous disease originating from post-thymus mature T lymphocytes, accounting for 5-20% of non-Hodgkin's lymphoma (NHL). In addition to extra nodal NK/T-cell lymphoma (ENKTL), nasal type, the most common subtypes of PTCL include peripheral T-cell lymphoma, not otherwise specified (PTCL, NOS), angioimmunoblastic T-cell lymphoma (AITL), anaplastic large cell lymphoma (ALCL), enteropathy-associated T-cell lymphoma (EATL) and monomorphic epitheliotropic intestinal T-cell lymphoma (MEITL). CHOP (cyclophosphamide, doxorubicin, vincristine and prednisone) or CHOP-like regimens are most commonly used first-line treatments in patients with PTCL. However, outcomes are poor with complete remission (CR) of about 50% and 5-year overall survival of only 25-45% 1,2. Although prognostic indicators such as the international prognostic index (IPI) and the prognostic index for T-cell lymphoma (PIT) have been proposed in PTCL, their prognostic values are not well established 3-6.

In recent years, a great deal of research evidences has supported the role of ^18^F-fluorodeoxyglucose positron emission tomography-computed tomography (^18^F-FDG PET/CT) in staging and response assessments of NHL. The prognostic value of ^18^F-FDG PET/CT quantitative parameters such as maximum standard uptake value (SUVmax), whole body metabolic tumor volume (WBMTV) and whole body total lesion glycolysis (WBTLG) have also been demonstrated in several studies. One study in South Korea suggested that high WBTLG was a better predictive factor for poor survival outcomes compared with IPI in diffuse large B-cell lymphoma (DLBCL) 7. WBSUVmax, a new prognostic model developed by our institution could also be a good predictor of the prognosis in ENKTL 8.However, to our knowledge, few studies have focused on the prognostic value of^ 18^F-FDG PET/CT quantitative parameters in PTCL. This study is aimed to investigate the prognostic value of PET/CT baseline quantitative parameters including SUVmax, WBSUVmax, WBMTV and WBTLG in patients with PTCL.

## Materials and Methods

### Clinical information

From January 2008 to May 2016, 80 patients with PTCL were enrolled in this study. Inclusion criteria were as follows: histologically confirmed PTCL including PTCL-NOS, AITL, ALCL and EATL; anthracycline-based regimen as first-line chemotherapy; PET/CT as initial evaluation. Patients with central nervous system involvement were excluded from this study.

### Instruments and methods

The procedure of ^18^F-FDG PET/CT examination was carried out as previously described, using Siemens Biograph 16 PET/CT HR scanner 8. A volume of interest (VOI) was set for each nodal or extra-nodal lesion by two nuclear medicine practitioners binded to patients' outcome. Quantitative parameters including SUVmax, metabolism tumor volume (MTV), total lesion glycolysis (TLG) were calculated automatically by the boundaries of voxels presenting SUV >3.0 (SUV3.0) on software (Planet Onco, version 2.0; DOSISoft). The inclusion and exclusion criteria of MTV were used in accordance with Kim et al's method 9. TLG=MTV×SUVmean in ROI. MTV/TLG of whole body lesions were added to obtain WBMTV/WBTLG. The SUVmax we recorded was the highest one of all lesions. WBSUVmax model presented by our center was the sum of the SUVmax of 11 nodal regions (Waldeyer ring, neck, infra-clavicular, axillary and pectoral, mediastinal, hilar, spleen, paraaortic, mesenteric, llilac, inguinal and femoral) and 10 extra-nodal regions (upper aero-digestive tract, skin/subcutaneous tissues, central nervous system (CNS) and spinal canal, lung, myocardium, bone and bone marrow, bowel, renal and adrenal, liver and testis) in whole body 8. As Liang et al reported that three WBSUVmax models, namely WB1SUVmax (Whole body SUVmax of 11 nodal and 10 extra-nodal regions), WB2SUVmax [Whole body SUVmax of 4 nodal (neck, axillary, inguinal and spleen) and 10 extra-nodal regions] and WBSUV3max [Whole body SUVmax of 3 nodal regions (superior diaphragm, inferior diaphragm and spleen) and 10 extra-nodal regions] could predict OS 8, we selected WBSUVmax3 as WBSUVmax model in this article due to its simplicity in calculation.

### Statistical analysis

Non-normal distribution data were described by median (range). The difference between the PET/CT quantitative parameters in the progression/non progression and survival/death subgroups was analyzed by Mann-Whitney *U* test. The optimal cut-off values for the PET/CT quantitative parameters were obtained by use of the receiver operating curve (ROC) analysis for OS. Differences between subgroups according to clinical characteristics were analyzed by Pearson χ2 test or Fisher exact test. Kaplan-Meier survival curve was used to estimate PFS and OS. Cox proportional hazards regression model were employed in multivariate analyses. Statistical analyses were done with software package SPSS 16.0. *P* <0.05 (two-sided) were considered statistically significant.

## Results

### Clinical characteristics

Clinical characteristics of patients are outlined in Table 1. 80 patients were enrolled in our study, including 29 patients with PTCL-NOS, 33 with AITL, 12with ALCL (6 with ALK positive) and 6 with EATL. The median age was 58 years (range, 17-89) and 44 patients were male. Seventy patients were presented with Ann Arbor stage III-IV, accounting for 87.5% of the total. Forty-nine patients had B symptoms. Sixteen patients had more than one extranodal site. Sixteen patients had a positive bone marrow biopsy. Forty-four patients had higher lactate dehydrogenase (LDH) than normal and 4 patients had hemophagocytic syndrome (HLH). Thirty-four patients had an IPI score of 3 to 5. All patients received CHOP, CHOP-like regimen or dose-adjusted etoposide, prednisone, vincristine, cysloposphamide and doxorubicin (DA-EPOCH) as first-line treatment. Median numbers of cycles per person were 6 (range, 4-8). Twenty-two patients received consolidative hematopoietic stem cell transplantation. With a median follow-up of 18 months (range, 5-73), 41 patients died, 13 patients survived with disease, and 26 patients survived without disease. The median PFS and OS were 13 months (95% CI 7.7-18.3) and 24 months (95% CI 17.2-30.8), respectively. Six patients with ALCL, ALK positive had a favorable prognosis compared with patients with other PTCL subtypes, with only one case had disease progression and died within 2 years, and the remaining 5 cases had been free of disease progression until the last follow-up.

### Comparison of the PET/CT metabolic quantitative parameters

**Figure 1:**
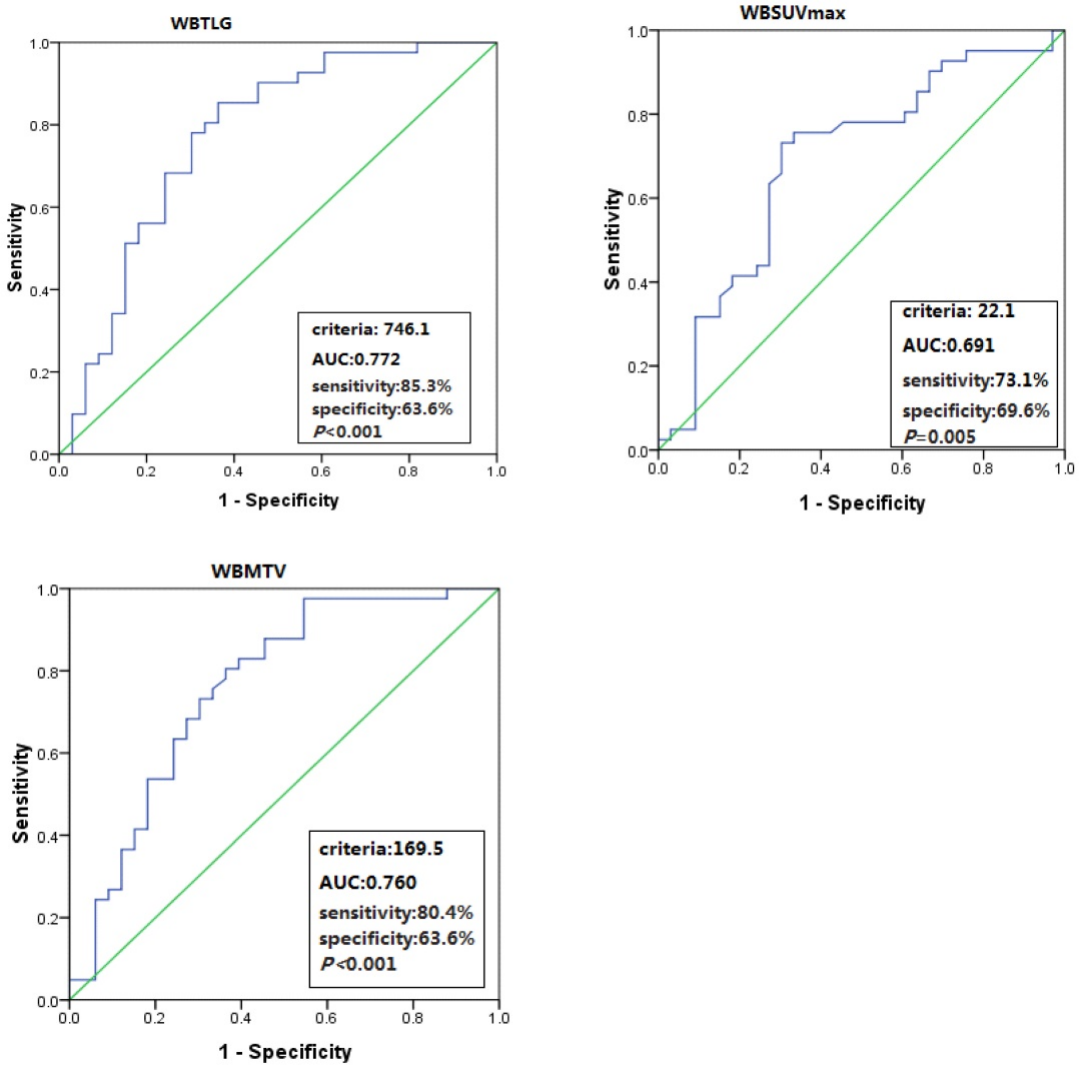
ROC curve analyses of WBSUVmax, WBMTV and WBTLG for OS.

The total population baseline PET/CT metabolic parameters were as follows: median SUVmax was 13.8 (range, 4.6-35.5), median WBSUVmax was 24.6 (range, 4.6-153.4), median WBMTV was 149 cm^3^ (range, 4-4545) and median WBTLG was 1017 (range, 16.5-23739). Six patients with ALCL, ALK positive were excluded in the following statistical analysis for their unique pathological types and good prognosis. Optimal cut-off values of WBSUVmax, WBMTV and WBTLG for OS were 22.2 (AUC=0.691; sensitivity 73.1%; specificity 69.6%; *P*=0.005), 169.5 cm^3^ (AUC=0.760; sensitivity 80.4%; specificity 63.6%; *P*<0.001) and 746.1 (AUC=0.772; sensitivity 85.3%; specificity 63.6%; *P*<0.001) in 74 patients with PTCL, respectively. Although the SUVmax optimal cut-off value was 9.7, the AUC in SUVmax was not significant for OS (Figure 1).

The population was dichotomized with the cut-off values of PET/CT metabolic parameters. PFS and OS did not differ significantly according to WBSUVmax, WBMTV and WBTLG. On the median PFS (12 months vs. 25 months, *P*=0.002; 12 months vs.44 months, *P*=0.008; 12 months vs.44 months; *P*=0.002) and the median OS (14 month vs. NR, *P*<0.001; 15 month vs. NR, *P*=0.001; 14 month vs. NR, *P*<0.001), there were significant differences in patients with a high and low WBSUVmax, WBMTV and WBTLG, respectively. SUVmax has no predictive value in both PFS and OS (Figure 2).

### Comparison of clinical and PET/CT parameters

Patient characteristics stratified according to cut-off values of PET/CT parameters are presented in Table 2. Patients with high WBSUVmax had more extranodal involvement, higher LDH level, IPI and PIT scores (*P*=0.016, 0.025, 0.001 and 0.009, respectively). High WBMTV was associated with high IPI and PIT scores, as well as advanced stage (*P*=0.024, 0.039 and 0.011, respectively). High WBTLG was corelated with advanced stage and high IPI scores (*P*=0.013 and 0.036, respectively). SUVmax was only corelated with IPI (*P*=0.024).

The results of univariate analyses for OS and PFS using the clinical variables and PET parameters were showed in Table 3. The variables significantly associated with both PFS and OS were WBSUVmax, WBMTV, WBTLG, Stage III-IV, B symptoms, ECOG PS≥2, BMB-positive, LDH, IPI, PIT. Extranodal sites>1 was predictive of shorter OS. Parameters significantly associated with PFS and OS were entered into multivariate Cox proportional hazards model. The results showed that only WBTLG >746.1 and high PIT were independent predictors of both shorter PFS and OS. The *P* value of stage III-IV with PFS was borderline.

## Discussion

PTCL is a group of highly aggressive diseases originating from post-thymus mature T lymphocytes. Although prognostic indices for PTCL such as IPI, PIT and IPTCLP have been developed, the prognostic values are not well defined. It has been reported that MTV and TLG of PET/CT could reflect tumor burden. Recently, MTV/TLG has been widely used for predicting survival and making therapeutic decisions in DLBCL 12-16. However, there have been few studies on the prognostic value of PET/CT parameters in PTCL. In these few studies, Deauville 5-point score (5-DS) of PET/CT was employed as a prognostic factor, but its prognostic value for PTCL has not been established. June* et al* 17 found that PET/CT 5-DS before treatment was predictive for prognosis in PTCL. Some other studies reported that PET/CT 5-DS after treatment could be a prognostic factor for PTCL 18,21. However, Gurion R *et al* 20 evaluated the prognostic value of P-PET/CT, I-PET/CT and E-PET/CT using 5-DS method in PTCL, and found that 5-DS of PET/CT could not predict the prognosis of patients with PTCL.

Compared with 5-DS, could MTV and TLG better predict the prognosis of patients with PTCL? A French multicenter study was conducted to analyze the prognostic value of baseline PET/CT quantitative parameters in PTCL. SUVmax of 41% was used as the ROI threshold in their study, and the cut-off value of MTV was 230 cm^3^ by X-tile analysis, which was significantly correlated with PFS and OS. The cut-off value of TLG was 1068, which was significantly correlated with PFS but was not statistically significant for OS. And there was no significant correlation between SUVmax and survival. In the correlation analysis of other clinical prognostic factors, MTV was significantly correlated with PIT and IPI. Multivariate survival analysis suggested that only MTV was an independent prognostic factor for PFS and OS 11.

Then, a further study of 140 patients with PTCL was carried out in seven research centers. PET/CT was performed after two cycles of treatment to evaluate the therapeutic effect using 5-DS method. They found that iPET/CT DS >3 could predict a poor prognosis. Combining 5-DS and MTV of PET/CT before treatment could better predict the prognosis of patients with PTCL 19.

To the best of our knowledge, our study is the first single center retrospective study to evaluate the prognostic value of baseline PET/CT quantitative parameters in the largest cohort of patients with PTCL. Among previous studies on PET/CT quantitative metabolic parameters in PTCL, ROI threshold was different. In South Korea, most of studies on ENKL used SUV 2.5 or SUV 3.0 as ROI threshold 7,9. We used SUV3.0 as the ROI threshold to calculate the WBMTV and WBTLG in 74 PTCL patients without 6 ALK^+^ALCL patients and the cut-off values were 169.5 cm^3^ and 746.1 by retrospective ROC analysis, respectively. Both parameters are significantly related to PFS and OS, which are not exactly the same as those in the French study.

The median SUVmax in our study was 13.8 (range, 4.6-35.5), suggesting that PTCL is a highly aggressive lymphoma. Similar to the study in France, our study did not found that the prognostic value of SUVmax, but WBSUVmax in our study was significantly associated with OS and PFS. The WBSUVmax used by our center was the sum of SUVmax of the whole-body lesions as previously reported and was significantly associated with the prognosis in NK/T cell lymphoma 8. WBSUVmax includes SUVmax of nodal and extra-nodal lesions, which could reflect the intensity of tumor invasion in patients. WBSUVmax eliminates the need for additional software and incorporates standard harmonization, making it more applicable than WBMTV and WBTLG in different research centers. Its prognostic value in ENKTL has also been demonstrated, suggesting that it might be an important prognostic indicator for lymphoma.

Our study further evaluated the correlations between four PET/CT quantitative parameters (SUVmax, WBSUVmax, WBMTV, WBTLG) and other clinical prognostic factors. Besides SUVmax, the other three quantitative parameters were also predictive factors of PFS and OS. We entered both image quantitative indicators and clinical indicators into unitivariate and multivariate analysis for PFS and OS. WBSUVmax, WBMTV, WBTLG and multiple clinical prognostic factors were significantly associated with prognosis in unitivariate analysis, while in multivariate analysis only WBTLG and PIT were independent prognostic factors. Our results demonstrate that combining WBTLG and PIT could better predict outcomes of patients with PTCL.

## Conclusions

Our study is one of the few studies focusing on the prognostic value of PET/CT quantitative parameters in PTCL. The results of our study suggest that high WBTLG, WBMTV and WBSUVmax could be prognostic factors of PTCL. WBSUVmax might be more applicable in clinical practice due to its simplicity in calculation. Combination of TLG and PIT could more accurately predict the prognosis of patients and guide treatments. Due to the relatively small sample size, further investigations are needed to confirm our findings.

## Figures and Tables

**Figrue 2:**
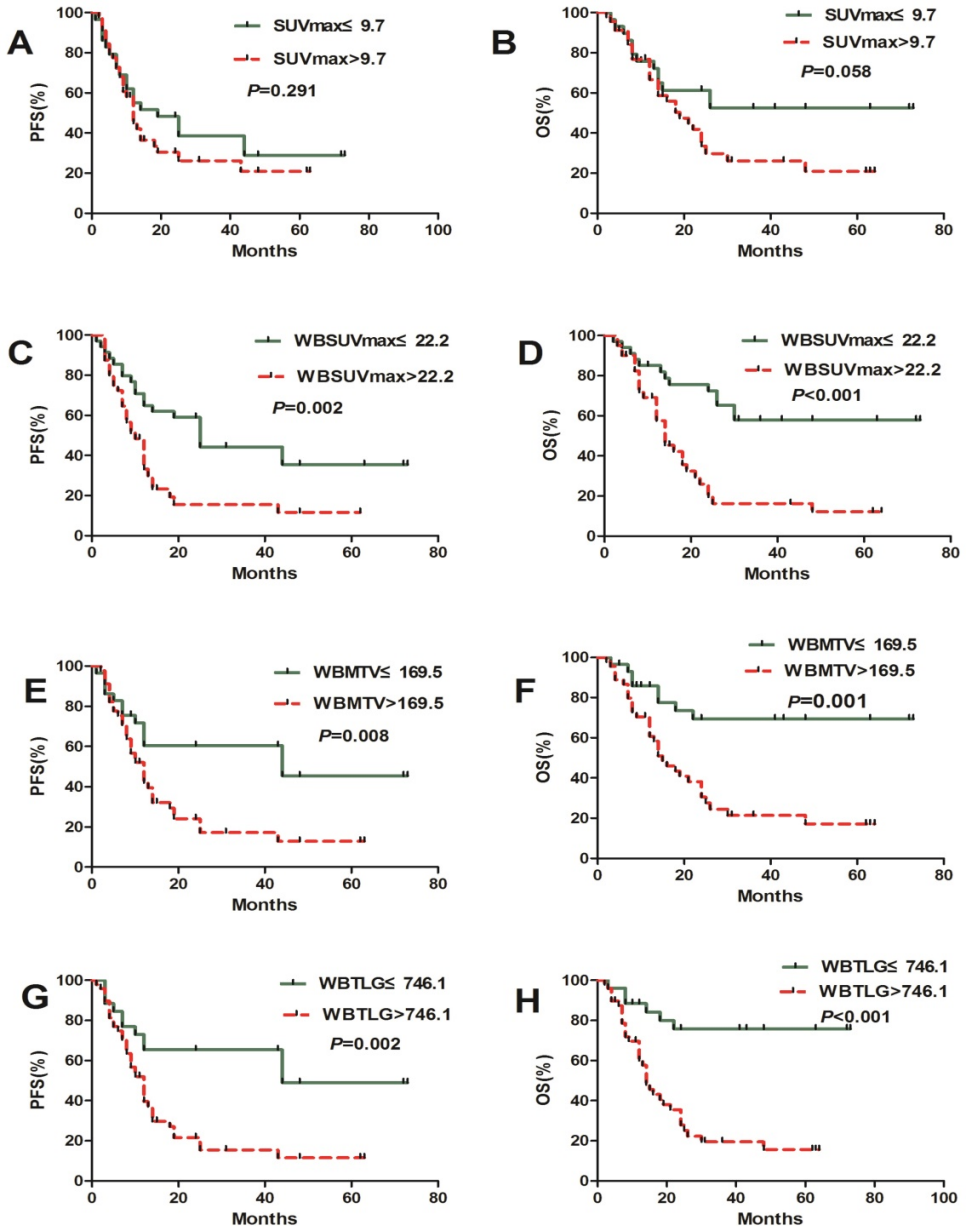
Kaplan-Meier estimates of PFS and OS according to the baseline SUVmax, WBSUVmax, WBMTV and WBTLG.

**Table 1:** Clinical characteristics for 80 patients with PTCL

Clinical characteristics	No. of patients	%
Age median (range)	58 (17-89)	
Sex (Male)	44	55
Histological type		
PTCL-NOS	29	36.2
AITL	33	41.3
ALK+ALCL	6	7.5
ALK‒ALCL	6	7.5
EATL	6	7.5
Ann Arbor stage (III-IV)	70	87.5
B symptoms	49	61.3
ECOG PS ≥2	16	20
Extranodal sites >1	22	27.5
BMB+	16	20
LDH >ULN	44	55
HLH	4	5.0
IPI score (3-5)	34	42.5
PIT score (2-4)	34	42.5
Consolidative transplant	22	27.5
Patient outcome		
No evidence of disease	26	32.5
Alive with disease	13	16.2
Death	41	51.3

Abbreviations: BMB^+^: positive bone marrow biopsy; ECOG PS: Eastern Cooperative Oncology Group performance status; HLH: hemophagocytic syndrome; IPI: international prognostic index; LDH: lactate dehydrogenase; PIT: the prognostic index for T-cell lymphoma; ULN: upper limit of normal

**Table 2:** Relationship with clinical characteristics and PET/CT parameters (74 PTCL patients without 6 ALK+ALCL patients)

Characteristics	SUVmax		*P*	WBSUVmax		*P*	WBMTV		*P*	WBTLG		*P*
	≤9.7 N=29	>9.7 N=45		≤22.2 N=34	>22.2 N=40		≤169.5 N=29	>169.5 N=45		≤746.1 N=26	>746.1 N=48	
Sex(M/F)	16/13	18/27	0.201	18/16	16/24	0.266	16/13	18/27	0.201	14/12	20/28	0.316
Age(≤60/>60)	18/11	24/21	0.459	21/13	21/19	0.423	17/12	25/20	0.795	17/9	25/23	0.270
Stage (I-II/III-IV)	5/24	4/41	0.283	6/28	3/36	0.330	7/22	2/43	0.011	7/19	2/46	0.013
B symptoms (No/Yes)	13/16	16/19	0.425	16/18	13/27	0.201	12/17	17/28	0.757	11/15	18/30	0.686
ECOG PS (<2/≥2)	23/6	35/10	0.876	30/4	28/12	0.058	23/6	35/10	0.876	21/5	37/11	0.713
Extranodal sites (≤1/>1)	24/5	29/16	0.088	29/5	24/16	0.016	24/5	29/16	0.088	21/5	32/16	0.199
BMB (Negtive/Positive)	23/6	35/10	0.876	29/5	29/11	0.183	23/6	35/10	0.876	20/6	38/10	0.823
LDH (≤ULN/>ULN)	14/15	17/28	0.379	19/15	12/28	0.025	15/14	16/29	0.169	13/13	18/30	0.298
HLH (No/Yes)	28/1	42/3	0.942	33/1	37/3	0.727	28/1	42/3	0.943	25/1	42/3	1.00
IPI score (0-2/3-5)	20/9	19/26	0.024	25/9	14/26	0.001	20/9	19/26	0.024	18/8	21/27	0.036
PIT score (0-1/2-4)	19/10	21/24	0.112	24/10	16/24	0.009	20/9	20/25	0.039	18/8	22/26	0.054

Abbreviations: BMB^+^: positive bone marrow biopsy; ECOG PS: Eastern Cooperative Oncology Group performance status; HLH: hemophagocytic syndrome; IPI: international prognostic index; LDH: lactate dehydrogenase; PIT: the prognostic index for T-cell lymphoma; SUVmax: maximum standard uptake value; ULN: upper limit of normal; WBMTV: whole body metabolic tumor volume; WBSUVmax: whole body SUVmax; WBTLG: whole body total lesion glycolysis

**Table 3:** Univariate and multivariate Cox regression analysis for PFS and OS.

Risk factors	Univariate (PFS)		Multivariate (PFS)		Univariate (OS)		Multivariate (OS)	
	HR (95%CI)	*P*	HR (95%CI)	*P*	HR (95%CI)	*P*	HR (95%CI)	*P*
**SUVmax >9.7**	1.365 (0.750-2.484)	0.308			1.879 (0.957-3.689)	0.067		
**WBSUVmax >22.2**	2.517 (1.357-4.670)	0.003			3.660 (1.812-7.392)	<0.001		
**WBMTV >169.5**	2.324 (1.202-4.492)	0.012			3.387 (1.561-7.349)	0.002		
**WBTLG >746.1**	2.877 (1.422-5.821)	0.003	2.130 (1.047-4.331)	0.037	4.759 (1.993-11.367)	<0.001	3.812 (1.579-9.204)	0.003
**Gender (Male)**	1.264 (0.705-2.269)	0.432			1.613 (0.852-3.053)	0.142		
**Age >60 years**	1.320 (0.741-2.354)	0.346			1.541 (0.833-2.851)	0.168		
**Stage III-IV**	11.582 (1.592-84.260)	0.016	7.128 (0.960-52.912)	0.055	8.878 (1.218-64.688)	0.031		
**B symptoms**	1.957 (1.055-3.630)	0.033			2.284 (1.142-4.566)	0.019		
**ECOG PS ≥2**	2.413 (1.259-4.625)	0.008			3.598 (1.787-7.243)	<0.001		
**Extranodal sites >1**	1.636 (0.895-2.989)	0.110			2.084 (1.112-3.906)	0.022		
**BMB-positive**	2.408 (1.251-4.639)	0.009			2.930 (1.142-4.566)	0.002		
**LDH >ULN**	2.123 (1.142-3.948)	0.017			3.105 (1.542-6.256)	0.002		
**HLH**	1.759 (0.627-4.936)	0.283			1.281 (1.542-6.256)	0.680		
**IPI 3-5**	2.193 (1.210-3.974)	0.010			3.599 (1.852-6.996)	<0.001		
**PIT 2-4**	2.649 (1.458-4.815)	0.001	2.078 (1.145-3.771)	0.016	4.305 (2.201-8.421)	<0.001	3.491(1.778-6.856)	<0.001

Abbreviations: Abbreviations: BMB^+^: positive bone marrow biopsy; ECOG PS: Eastern Cooperative Oncology Group performance status; HLH: hemophagocytic syndrome; IPI: international prognostic index; LDH: lactate dehydrogenase; PIT: the prognostic index for T-cell lymphoma; SUVmax: maximum standard uptake value; ULN: upper limit of normal; WBMTV: whole body metabolic tumor volume; WBSUVmax: whole body SUVmax; WBTLG: whole body total lesion glycolysis.

## References

[1] Broussais-Guillaumot F, Coso D, Belmecheri N (2013). Peripheral T-cell lymphomas: analysis of histology, staging and response to treatment of 208 cases at a single institution. Leuk Lymphoma.

[2] Mak V, Hamm J, Chhanabhai M (2013). Survival of patients with peripheral T-cell lymphoma after first relapse or progression: spectrum of disease and rare long-term survivors. J Clin Oncol.

[3] Weisenburger DD, Savage KJ, Harris NL (2011). International Peripheral T-cell Lymphoma Project. Peripheral T-cell lymphoma, not otherwise specified: a report of 340 cases from the International Peripheral T-cell Lymphoma Project. Blood.

[4] Went P, Agostinelli C, Gallamini A (2006). Marker expression in peripheral T-cell lymphoma: a proposed clinical pathologic prognostic score. J Clin Oncol.

[5] Gallamini A, Stelitano C, Calvi R (2004). Peripheral T cell lymphoma unspecified (PTCL-U): a new prognostic model from a retrospective multicentric clinical study. Blood.

[6] Savage KJ, Harris NL, Vose JM (2008). ALK- anaplastic large-cell lymphoma is clinically and immunophenotypically different from both ALK+ ALCL and peripheral T-cell lymphoma, not otherwise specified: report from the International Peripheral T-Cell Lymphoma Project. Blood.

[7] Kim TM, Paeng JC, Chun IK (2013). Total lesion glycolysis in positron emission tomography is a better predictor of outcome than the international prognostic index for patients with diffuse large B cell lymphoma. Cancer.

[8] Liang JH, Ding CY, Gale RP (2017). Prognostic value of whole-body SUVmax of nodal and extra-nodal lesions detected by 18F-FDG PET/CT in extra-nodal NK/T-cell lymphoma. Oncotarget.

[9] Kim CY, Hong CM, Kim DH (2013). Prognostic value of whole-body metabolic tumour volume and total lesion glycolysis measured on 18F-FDG PET/CT in patients with extranodal NK/T-cell lymphoma. Eur J Nucl Med Mol Imaging.

[10] Song MK, Chung JS, Shin HJ (2013). Clinical value of metabolic tumor volume by PET/CT in extranodal natural killer/T cell lymphoma. Leuk Res.

[11] Cottereau AS, Becker S, Broussais F (2016). Prognostic value of baseline total metabolic tumor volume (TMTV0) measured on FDG-PET/CT in patients with peripheral T-cell lymphoma (PTCL). Ann Oncol.

[12] Adams HJ, de Klerk JM, Fijnheer R (2015). Prognostic superiority of the National Comprehensive Cancer Network International Prognostic Index over pretreatment whole-body volumetric-metabolic FDG-PET/CT metrics in diffuse large B-cell lymphoma. Eur J Haematol.

[13] Cottereau AS, Lanic H, Mareschal S (2016). Molecular Profile and FDG-PET/CT Total Metabolic Tumor Volume Improve Risk Classification at Diagnosis for Patients with Diffuse Large B-Cell Lymphoma. Clin Cancer Res.

[14] Mikhaeel NG, Smith D, Dunn JT (2016). Combination of baseline metabolic tumour volume and early response on PET/CT improves progression-free survival prediction in DLBCL. Eur J Nucl Med Mol Imaging.

[15] Sasanelli M, Meignan M, Haioun C (2014). Pretherapy metabolic tumour volume is an independent predictor of outcome in patients with diffuse large B-cell lymphoma. Eur J Nucl Med Mol Imaging.

[16] Schöder H, Zelenetz AD, Hamlin P (2016). Prospective study of 3′-deoxy-3′-18F-fluorothymidine PET for early interim response assessment in advanced-stage B-cell lymphoma. J Nucl Med.

[17] Jung SH, Ahn JS, Kim YK (2015). Prognostic significance of interim PET/CT based on visual, SUV-based, and MTV-based assessment in the treatment of peripheral T-cell lymphoma. BMC Cancer.

[18] El-Galaly TC, Pedersen MB, Hutchings M (2015). Utility of interim and end-of-treatment PET/CT in peripheral T-cell lymphomas: A review of 124 patients. Am J Hematol.

[19] Cottereau AS, El-Galaly TC, Becker S (2018). Predictive Value of PET Response Combined with Baseline Metabolic Tumor Volume in Peripheral T-Cell Lymphoma Patients. J Nucl Med.

[20] Gurion R, Bernstine H, Domachevsky L (2018). Utility of PET-CT for Evaluation of Patients With Peripheral T-cell Lymphoma. Clin Lymphoma Myeloma Leuk.

[21] Yhim HY, Park Y, Han YH (2018). A risk stratification model for nodal peripheral T-cell lymphomas based on the NCCN-IPI and posttreatment Deauville score. Eur J Nucl Med Mol Imaging.

